# Leap motion controlled video game-based therapy for upper limb rehabilitation in patients with Parkinson’s disease: a feasibility study

**DOI:** 10.1186/s12984-019-0593-x

**Published:** 2019-11-06

**Authors:** Pilar Fernández-González, María Carratalá-Tejada, Esther Monge-Pereira, Susana Collado-Vázquez, Patricia Sánchez-Herrera Baeza, Alicia Cuesta-Gómez, Edwin Daniel Oña-Simbaña, Alberto Jardón-Huete, Francisco Molina-Rueda, Carlos Balaguer-Bernaldo de Quirós, Juan Carlos Miangolarra-Page, Roberto Cano-de la Cuerda

**Affiliations:** 1 Department of Physical Therapy, Occupational Therapy, Physical Medicine and Rehabilitation. Faculty of Health Sciences, Rey Juan Carlos University, Avenida de Atenas s/n 28922 Alcorcón, Madrid, Spain; 2 Robotics Lab, University Carlos III of Madrid, Leganés, Madrid, Spain; 3 Chair of Rehabilitation Unit, Hospital Universitario de Fuenlabrada, Fuenlabrada, Madrid, Spain

**Keywords:** Virtual reality, Non-immersive video games, Leap motion controller, Parkinson’s disease, Upper limb, Dexterity

## Abstract

**Background:**

Non-immersive video games are currently being used as technological rehabilitation tools for individuals with Parkinson’s disease (PD). The aim of this feasibility study was to evaluate the effectiveness of the Leap Motion Controller® (LMC) system used with serious games designed for the upper limb (UL), as well as the levels of satisfaction and compliance among patients in mild-to-moderate stages of the disease.

**Methods:**

A non-probabilistic sampling of non-consecutive cases was performed. 23 PD patients, in stages II-IV of the Hoehn & Yahr scale, were randomized into two groups: an experimental group (*n* = 12) who received treatment based on serious games designed by the research team using the LMC system for the UL, and a control group (*n* = 11) who received a specific intervention for the UL. Grip muscle strength, coordination, speed of movements, fine and gross UL dexterity, as well as satisfaction and compliance, were assessed in both groups pre-treatment and post-treatment.

**Results:**

Within the experimental group, significant improvements were observed in all post-treatment assessments, except for Box and Blocks test for the less affected side. Clinical improvements were observed for all assessments in the control group. Statistical intergroup analysis showed significant improvements in coordination, speed of movements and fine motor dexterity scores on the more affected side of patients in the experimental group.

**Conclusions:**

The LMC system and the serious games designed may be a feasible rehabilitation tool for the improvement of coordination, speed of movements and fine UL dexterity in PD patients. Further studies are needed to confirm these preliminary findings.

## Introduction

The second most common neurodegenerative disorder, after Alzheimer’s disease, is Parkinson’s disease (PD), which is prevalent in approximately 1% of people aged 60 years or older [1, 2]. This disorder, which predominately impairs motor function, affects 1–5% of individuals aged 65–69 years of age and 1–3% of those above 80 years of age. The cardinal symptoms are: *bradykinesia*, defined in part by James Parkinson as being “lessened muscular power”, and which manifests as slowness of movement; *rigidity,* defined as an increased muscular tone when the limb is passively moved and which is usually experienced as a sense of feeling stiff and uncomfortable; *resting tremor,* defined as a repetitive back-and-forth movement of any limb, which occurs when that part of the body is not actively moving; and *postural instability:* which refers to an impaired reaction when balance is perturbed. Additionally, patients with PD typically suffer from a wide range of motor and non-motor problems [3]. These signs and symptoms impair the performance of their daily activities, reducing their level of independence. At present, there is no curative treatment for PD, rather, treatments are focused on the symptoms and prevention of the progression of the disease [4, 5].

Throughout the various stages of PD, impaired dexterity is among the most frequently reported disturbing symptom and a major contributor to the burden of the disease [6]. Dexterity deficits impair typical activities of daily living and may be present even in mild to moderate stages of PD. Patients with PD become dependent on caregivers because their motor and cognitive disabilities interfere with their ability to perform daily activities [6].

Scientific evidence to date supports the benefits of rehabilitation treatment in PD [7, 8]. In the field of neurorehabilitation, technology-based rehabilitation systems, such as virtual reality (VR), are promising and may be able to deliver a client-centered task-oriented rehabilitation. Several studies have addressed the positive effects of VR systems as being a complementary therapy to neurological rehabilitation [9]. These systems are based on computer-based technology that allows users to interact with simulated environments and receive feedback on performance within real-time scenarios, therefore providing the opportunity to perform functional and repetitive activities, facilitating motor learning and neuroplasticity through increased intensity during task-oriented training [9].

Video games based on VR technology are emerging as valid tools used in neurorehabilitation for patients with neurological disorders, and as a low cost and easily accepted adjunct to traditional therapy. Standard games such as the Nintendo Wii, Playstation Move and Kinect plus XBOX 360 have been used in PD rehabilitation. However, often these are either too difficult for patients or the games progress too quickly, failing to provide impairment-focused training or specifically address patients’ needs [10]. Therefore, it is necessary to develop specific serious games for PD patients. Serious games are defined as games designed for a primary purpose other than that of pure entertainment, and which promote learning and behavior changes for PD patients.

In this context, new low-cost markerless devices have emerged, such as the Leap Motion Controller (LMC) System®, which uses a sensor that captures the movement of the patient’s forearms and hands without the need to place sensors or devices on the body. This generates a virtual image of the upper limbs on a computer screen and the patient is prompted to perform movements according to the functional task proposed. This system presents important advantages over other motion capture systems, namely thanks to its portability, ease of use, commercial availability, low cost and non-invasive nature. However, evidence is lacking that supports the therapeutic use of LMC in the treatment of upper limb (UL) motor disorders in PD. Furthermore, to our knowledge, no specific serious games have been designed for PD patients using the LMC system.

Therefore, the primary aim of the present study was to evaluate the effectiveness of the LMC system using serious games designed for improving UL grip muscle strength, coordination, speed of movements and fine and gross dexterity. Furthermore, we sought to assess satisfaction and compliance levels among those in mild-to-moderate stages of the disease.

## Materials and methods

### Participants

All patients were recruited from the Association of Patients with PD ) (Aparkan) in Alcorcón (Madrid, Spain). Non-probabilistic sampling of non-consecutive cases was performed.

The inclusion criteria were: patients with PD who fulfilled the modified diagnostic criteria of the Brain Bank of the United Kingdom; patients in stages II, III and IV of the Hoehn & Yahr scale; > 60% Schwab & England functionality scale; patients whose motor response to pharmacological treatment was stable or slightly fluctuating, and who were not receiving specific UL rehabilitation treatment at the time of the study.

The study exclusion criteria were: the diagnosis of diseases other than PD or serious injuries affecting the UL; the inability to understand instructions and actively cooperate in the tasks indicated based on a score ≥ 24 in the Mini-mental Test; refusal to participate in the study; stages I or V of the Hoehn & Yahr scale; and visual impairment not correctable by glasses.

### Procedure

The sample was randomized into two groups: an experimental group, who received UL treatment based on serious games designed by the research team, using the LMC system; and a control group, who received a specific UL intervention based on conventional physical therapy (based on shoulder, elbow, wrist and finger mobilization, strengthening of UL extensor muscles, stretching exercises for UL flexor muscles) [7, 8] and with functional task practice trying to imitate the movements of the serious games designed for the experimental group (− i.e. reaching movements, dexterity, grasping and pincer grasp movements using objects of daily living, such as coins, keys, balls, cups, plates-).

This protocol was approved by the local ethics committee of the Rey Juan Carlos University. Informed consent was obtained from all participants included in this study.

All groups received the intervention at the Aparkan Association, between May and July of 2017. Both the experimental group and the control group received two 30 min sessions per week over a six-week period (a total of 12 sessions for each group). A physical therapist was present throughout the process. The experimental group used the LMC system while seated at a table placed at mid-trunk height and with the elbow placed at an initial 90° elbow flexion. When necessary, manual assistance by the physical therapist was provided on the patient’s most affected side.

**Fig. 1:**
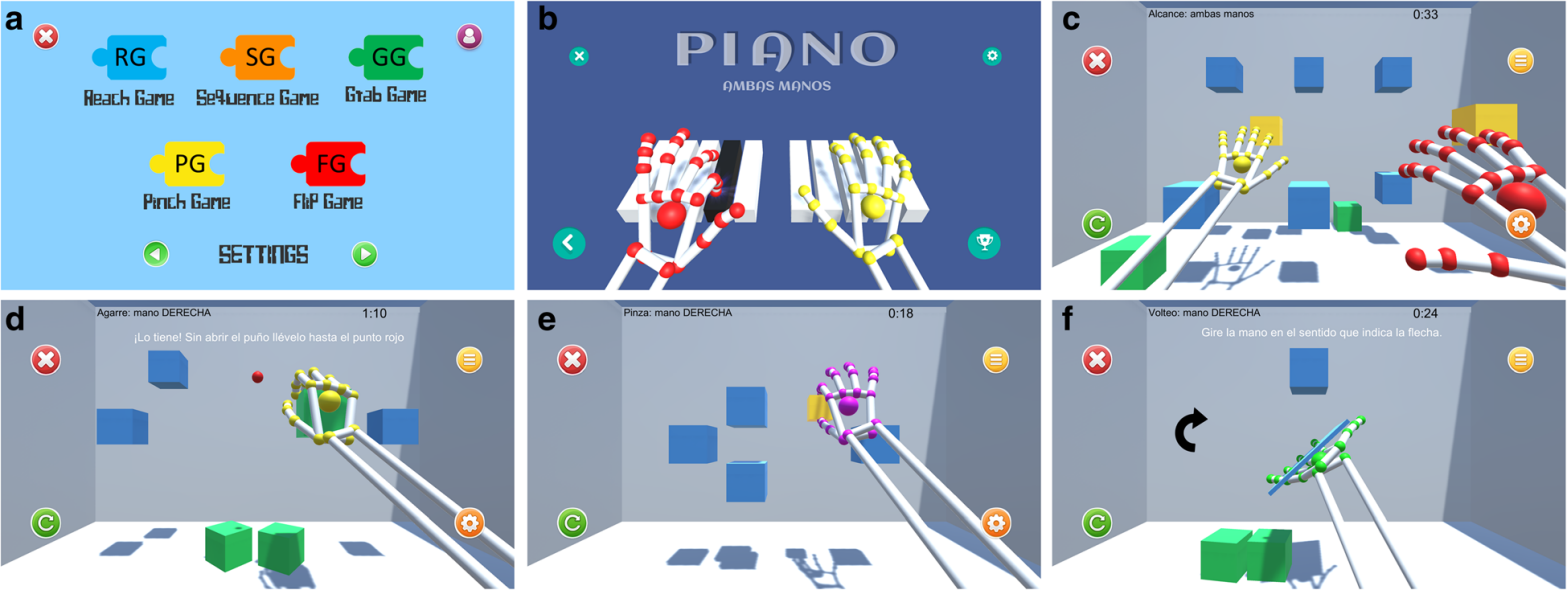
Serious games designed for the Leap Motion® System. *Serious games used on protocol: **a**) Games Menu, **b**) The Piano Game, **c**) The Reach Game, **d**) The Grasp Game, **e**) The Pinch Game, and **f**) The Flip Game

The serious games performed in this study aimed to imitate exercises and movements commonly included in conventional rehabilitation, such as palmar prehension, finger flexion and extension or hand pronation-supination (Fig. 1). Patients performed six games: the Piano Game (PI), the Reach Game (RG), the Sequence Game (SG), the Grasp Game (GG), the Pinch Game (PG) and the Flip Game (FG). Each of these games was based on a different rehabilitation goal.

### Description of the video games

**Fig. 2:**
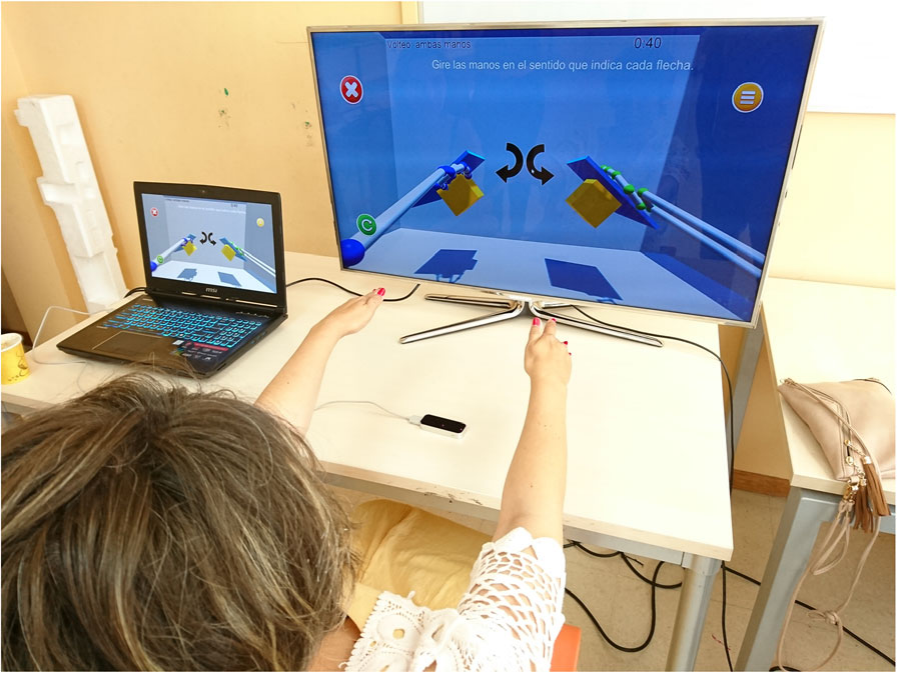
A Parkinson’s disease patient practicing a video game based on cubes (Flip Game)

A set of video games was developed, aimed at UL motor rehabilitation. The Leap Motion sensor was used to capture the users’ hand movements and different virtual environments were created using Unity3D Game Engine software. In total, six video games were developed: the PI, the RG, the SG, the GG, the PG and the FG. Each game focused on different rehabilitation purposes, based on requirements and guidelines suggested by clinical experts on PD neurorehabilitation. The games were performed firstly unilaterally (each hand separately) and then bilaterally (both hands at the same time). The user interface allows therapists and patients to easily navigate through the games. For this purpose, the instructions are given clearly and precisely via texts and audio cues. It has been described that individuals with PD may move more quickly or easily when their actions are in response to environmental stimuli (i.e., exogenously evoked) than when their actions are spontaneous and self-initiated (i.e., endogenously evoked) [10], so using this task switching paradigm, visual and acoustic cues were given to the patients to incite the specific movements on each game. A full description of these games is provided in a previous study [11]. However, the main features and procedures of the video games are described below:

- PI: This video game features a virtual piano keyboard with ten keys, each corresponding to a single finger on each hand (see Fig. 1b). The user is encouraged to play each piano key with the corresponding finger. During the game, the required key to be pressed lights up. The keys are lit up first in an ordered sequence, from the little finger to the thumb, and then in a random sequence. Each key that is correctly pressed is recorded and a point is added to the score. Higher scores equal better performance of the game.
- RG: In this game, several cubes are shown in different spatial positions, placed within the reaching range of the user’s upper extremity (see Fig. 1c). A highlighted cube indicates the target to be touched. When the user reaches the cube, it falls to the floor of the virtual scene. To complete the game, the user must reach all cubes.
- SG: This game uses the same set-up as the Reach Game. A sequence of cubes is presented to the user, who must memorize the sequence and repeat it by reaching the cubes in the same order shown.
- GG: This game encourages the user to perform finger flexion and extension movements, similar to grasping movements. A series of cubes are shown, including a red circle in the center of the screen (see Fig. 1d). When a cube is highlighted, the user must grasp the cube and move it to the red circle while keeping their fist closed. The cube may only be released when it touches the red circle.
- PG: The purpose of this game is to train bidigital grip via the performance of a pinching movement between the thumb and the index fingers. As in the previously explained games, a set of spatially distributed cubes are presented to the user, (see Fig. 1e). When a cube is highlighted, the user must place their hand near the target cube and make the cube smaller, using a pinching movement, until the cube disappears.
- FG: This game trains pronation and supination movements of the forearm. The user must place the palm of the hand over the Leap Motion device imitating a waiter holding out a tray (Fig. 1f). A small tray with a cube in the middle appears in the center of the screen. The patient should then turn the palm downwards. Upon doing so, the cube detaches from the tray and falls to the ground (Fig. 2).

The games are easy to customize according to the patients’ needs and skill level. The settings can be defined by therapists at the beginning of the training session, or during the performance of the video game. The physical appearance of the piano keyboard can be adjusted by using slider controls in order to better accommodate the game to each patient. These sliders are used to modify the keyboard properties, such as the distance between keys (defining the degree of dissociation between fingers), the width of each key (allowing a large or small contact area), the height required for pressing each button (depth that the user must push the key), or the keyboard height. The latter is a particularly relevant feature as it allows therapists to first identify the optimal hand position (the hands are placed in the air over the device) that the user is comfortable with, and then the keyboard can be moved up or down until it is in contact with the virtual hands.

Overall, the remaining games (Reach Game, Sequence Game, Grab Game, Pinch Game, and Flip Game) can also be adjusted for performance and appearance. The settings options include: (1) the number of cubes, which is related to the number of repetitions of each task; (2) the size of the cubes, by choosing among small, medium, or large sizes; and (3) the number of cubes for users to remember during the Sequence Game.

The information obtained in each session can be automatically stored in the patient’s record in a format that medical staff can easily handle in order to perform their evaluations. In this way, CSV files easily match the specifications required and its content can be effortlessly managed. Conversely, it is possible to access an updated report of each patient, allowing the physician to remotely supervise the patient’s progress. The record of each patient is identified by a code, to guarantee privacy.

**Fig. 3:**
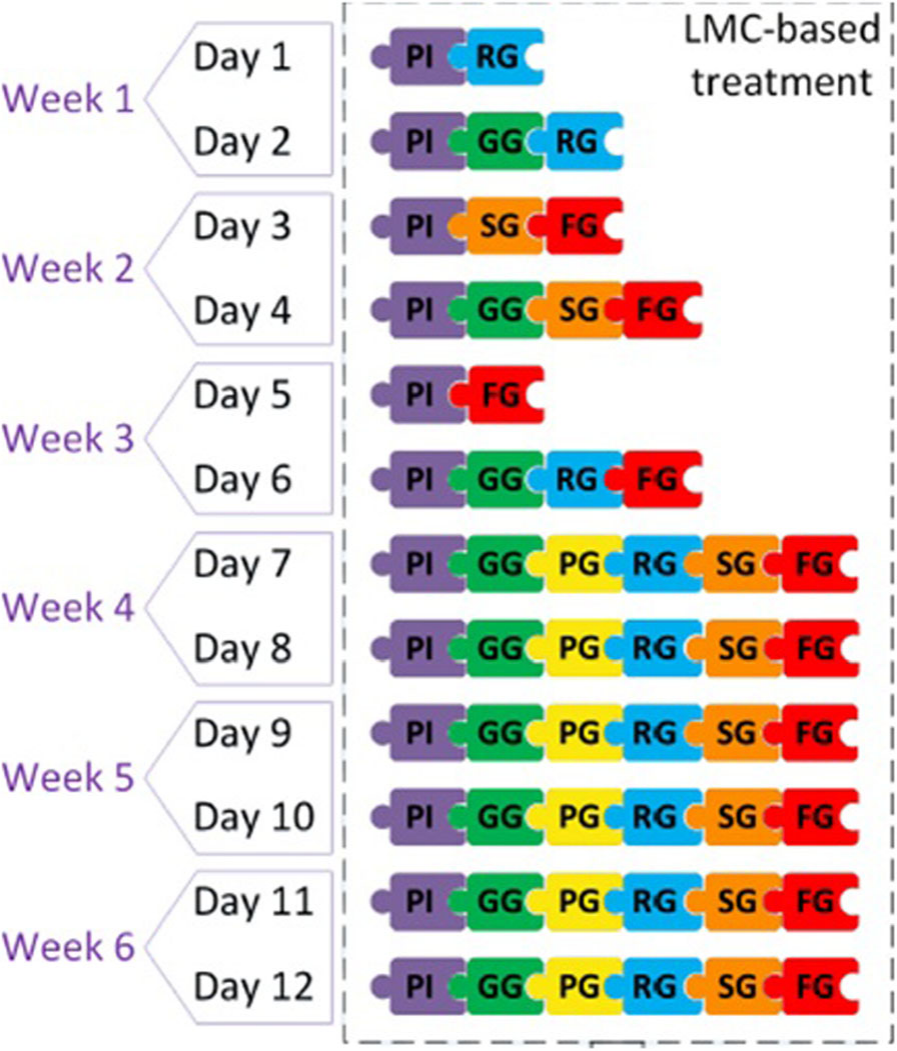
Experimental protocol. *PI: Piano Game; GG: Grasp Game; PG: Pinch Game; RG: Reach Game; SG: Sequence Game; and FG: Flip Game

Therefore, different interventions can be designed by combining two or more games that focus on a specific pathology and patient population. The protocol used in this study is shown in Fig. 3. As the patient progresses, the difficulty and number of the exercises increases. Rest periods are built in depending on the individual patients’ needs.

All measurements were performed at the Movement Analysis Laboratory located at the Health Sciences Faculty of the XX University. Two evaluations were conducted: pre-treatment and post-treatment. The intervention and all tests were performed within two hours of administration of anti-Parkinsonian medication, during the “on” phase of the medication cycle, as this is the period during which patients perform most of their daily activities.

### Outcome measures

A Jamar® hydraulic hand dynamometer was used to measure grip strength. This dynamometer offers accurate and repeatable grip strength readings scaled in pounds and kilograms. All the patients performed three grip movements, and the mean values were recorded. The data for the less and more affected sides were recorded in kilograms. The Jamar® hydraulic hand dynamometer is one of the most used objective tools to assess grip strength, being considered a device of excellent reliability, sensitive, and ease of use. It is recommended by the American Society of Hand Therapists and by the Brazilian Society of Hand Therapists [12].

The Box and Blocks Test (BBT) was performed to measure unilateral gross manual dexterity in both the less and more affected side. The BBT consists of moving the maximum number of blocks from one compartment of a box to another, one by one, within one minute. The BBT is a quick, simple, and reliable measurement of manual dexterity. Its administration procedure is standardized and its validity has been shown in elderly subjects with upper limb disability [13, 14].

The Purdue Pegboard Test (PPT) was used to assess coordination, speed of movement and fine motor dexterity. The PPT features a board with two columns with 25 holes each and a specific number of pins, washers and collars placed in four containers across the top of the board. The test consists of inserting as many pins as possible in three distinct phases, with a time limit of 30 s for each. First, the test is performed with the less affected side, then with the more affected side, then with both hands at the same time and, finally, an assembly test is performed (60 s). The number of pins inserted is subsequently recorded. The PPT is a reliable assessment to evaluate manual dexterity in PD patients [15, 16].

The Client Satisfaction Questionnaire (CSQ-8) evaluates the satisfaction of health service users. This is a self-administered post-treatment questionnaire, comprising eight items which evaluate the level of satisfaction regarding the care and quality of the service received and the level of fulfillment of the patient’s expectations regarding the treatment administered. The total score of the questionnaire is 32 points, with higher values meaning higher satisfaction with the treatment received [17, 18].

Additionally, we recorded the attendance rate (%) for therapy sessions (compliance).

### Statistical analysis

The statistical analysis was performed using the SPSS statistical software system (SPSS Inc., Chicago, IL; version 22.0). The Shapiro Wilk’s test and the Kolmogorov-Smirnov test were used to screen all data for normality of distribution. Additionally, the Wilcoxon test for related samples and the Mann-Whitney test for non-related samples were used for to compare variables. The statistical analysis was performed with a 95% confidence level, and significant values were considered as *p* < 0.05. We used the mean and the standard deviation of parameters to calculate de effect size for the comparisons using the Cohen’s d statistic. Mean differences of 0.2, 0.5, and 0.8 standard deviations are considered ‘small’, ‘medium’, and ‘large’ effect sizes respectively.

## Results

The sample consisted of a total of 23 patients, 11 male and 12 female, of the 26 selected at the study onset. Three subjects were excluded due to an inability to attend the assessment and/or treatment sessions. The age of the patients ranged from 45 to 79 years (mean age 66.65 ± 10.14 years). In 15 patients, the more affected side was on the left, whereas the right side was the most affected for the remaining eight patients. The Schwab and England scores of patients ranged from 100 to 60% of independence (73.50 ± 12.25%). The patients were randomly assigned into two groups, 12 of whom were assigned to the experimental group while 11 were assigned to the control group (Table 1). Within-group and intergroup statistical analysis are summarized in Tables 2 and 3.

**Table 1:** Patient features

Groups (n)	Age (years) Mean (±Standard deviation)	Gender	Hoenhn & Yahr	More affected side	Schwab and England score (%) Mean (±Standard deviation)
Experimental group (12)	65.77 (±7.67)	6 Male 6 Female	II (5) III (6) IV (1)	3 Right 9 Left	73.33 (±12.24)
Control group (11)	67.36 (±12.12)	5 Male 6 Female	II (6) III (4) IV (1)	5 Right 6 Left	73.63 (±12.86)

**Table 2:** Outcome scores (experimental and control groups)

Variable			Experimental group		Control group	
			Median (IR)	*p*-value	Median (IR)	*p*-value
Jamar	More affected	Pre	14.66 (9.00)	.003*	18.66 (14.66)	.123
		Post	27.33 (17.33)		19.66 (12.83)	
	Less affected	Pre	19.33 (15.67)	.005*	20.00 (11.50)	.944
		Post	26.33 (28.00)		24.00 (9.67)	
BBT	More affected	Pre	42.00 (23.00)	.014*	39.00 (17.50)	.293
		Post	46.00 (12.00)		45.00 (8.50)	
	Less affected	Pre	46.00 (26.00)	.090	48.00 (16.00)	.141
		Post	49.00 (13.00)		49.00 (11.00)	
PPT	More affected	Pre	8.00 (4.33)	.003*	8.66 (3.67)	.024*
		Post	12.33 (8.33)		9.66 (3.00)	
	Less affected	Pre	9.00 (5.00)	.009*	10.00 (3.50)	.248
		Post	11.66 (5.00)		10.50 (2.50)	
PPT both hands		Pre	8.66 (3.33)	.005*	10.66 (7.67)	.722
		Post	10.33 (8.00)		12.00 (6.33)	
PPT assembly		Pre	12.66 (13.66)	.003*	14.66 (7.67)	.237
		Post	23.66 (13.67)		16.00 (4.17)	

*BBT* box and block test, *PPT* Purdue Pegboard Test. Data are expressed as median and interquartile range (IR). *p value < 0.05 using the Wilcoxon test for related samples

**Table 3:** Comparison of outcome scores between the experimental group and the control group

Variable			Median (Interquartile range)		p-value
			Experimental group	Control group	
Pre	Jamar	More affected	14.66 (9.00)	18.66 (14.66)	.648
		Less affected	19.33 (15.67)	20.00 (11.50)	1.000
	BBT	More affected	42.00 (23.00)	39.00 (17.50)	.424
		Less affected	46.00 (26.00)	48.00 (16.00)	.909
	PPT	More affected	8.00 (4.33)	8.66 (3.67)	.819
		Less affected	9.00 (5.00)	10.00 (3.50)	.879
	PPT both hands		8.66 (3.33)	10.66 (7.67)	.447
	PPT assembly		12.66 (13.66)	14.66 (7.67)	.790
Post	Jamar	More affected	27.33 (17.33)	19.66 (12.83)	.087
		Less affected	26.33 (28.00)	24.00 (9.67)	.210
	BBT	More affected	46.00 (12.00)	45.00 (8.50)	.381
		Less affected	49.00 (13.00)	49.00 (11.00)	.518
	PPT	More affected	12.33 (8.33)	9.66 (3.00)	.036*
		Less affected	11.66 (5.00)	10.50 (2.50)	.447
	PPT both hands		10.33 (8.00)	12.00 (6.33)	.879
	PPT assembly		23.66 (13.67)	16.00 (4.17)	.006*

*BBT* box and block test, *PPT* Purdue Pegboard Test. Data are expressed as median and interquartile range. *p value < 0.05 using Mann-Whitney test for not related samples

The within-group statistical analysis for the experimental group showed significant improvements in all post-treatment assessments, except for the BBT on the less affected side. Significant improvements were observed on the Jamar for the more affected side (*p* = .003) and the less affected side (*p* = .005); the BBT for the more affected side (*p* = .014); the PPT for the more affected side (p = .003), the PPT for the less affected side (*p* = .009), the PPT both hands (p = .005) and the PPT assembly (p = .003) (Table 2). The effect size was large (>.80) for Jamar (more affected side) and PPT assembly; and medium (>.50) for PPT (both sides) (Table 4). Clinical improvements were observed for all assessments in the control group, but statistical significance was only reached for the PPT on the more affected side (*p* = .024) (Table 3).

**Table 4:**
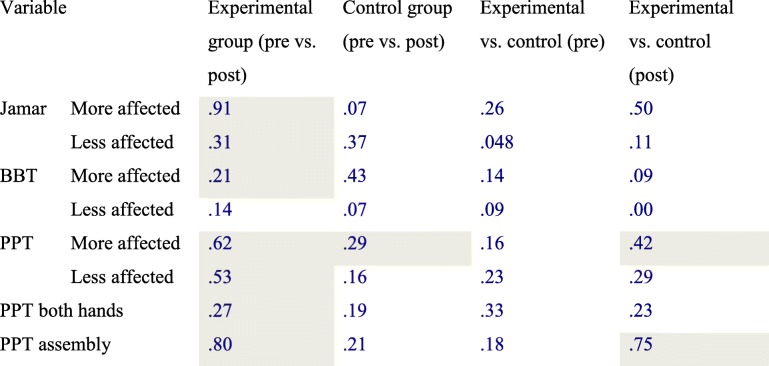
The effect size estimators for the comparisons

Cells in gray are differences with statistical significance

According to the statistical intergroup analysis, no significant difference was observed between either of the two groups in terms of baseline clinical characteristics. In the experimental group, significant improvements were found for the PPT on the more affected side (*p* = .036) and the PPT assembly (*p* = .006) post-treatment, when compared to the control group (Table 4). The effect size was large (>.80) for the PPT assembly (Table 4).

The CSQ-8 showed a high degree of satisfaction for both groups. The experimental group obtained a mean of 29.6 (1.51) points and the control group obtained a mean of 28.75 (.5) points out of the maximum of 32. Of the eight items considered by this questionnaire, the entire sample gave the maximum score in response to questions N° 4 (If a friend were in need of similar help, would you recommend our program to him or her?) and N° 7 (In general, are you satisfied with the services you have received?). The experimental group also gave the maximum score for N° 1 (How do you evaluate the quality of the service you received?) and the control group also gave the maximum score for N° 5 (Are you satisfied with the help you have received?) and N°8 (If you were to seek help again, would you come back to our program?). None of the participants expressed disagreement or dissatisfaction in response to the remaining questions (Table 5).

**Table 5:** The Client Satisfaction Questionnaire (CSQ-8)

Variable	Experimental group	Control group
1. Quality of service	4 (0)	3 (0)
2. Kind of service	3.4 (.54)	3 (0)
3. Met need	3.2 (.44)	3.5 (.57)
4. Recommend to a friend	4 (0)	4 (0)
5. Amount of help	3.8 (.44)	4 (0)
6. Deal with problems	3.4 (.54)	3.25 (.5)
7. Overall satisfaction	4 (0)	4 (0)
8. Come back	3.8 (.44)	4 (0)
Total Score	29.6 (1.51)	28.75 (.5)

Data are expressed as mean and standard deviation

Furthermore, compliance to the interventions was excellent (100%) and no adverse side-effects were observed for both groups.

## Discussion

Parkinson’s disease affects millions of people worldwide. Since the disease strongly influences the quality of life of patients, raising the burden of care and the costs for society, optimal solutions for the treatment of PD are needed [9, 19]. Serious games based on the LMC system present promising tools for UL neurorehabilitation in people with PD. The purpose of this study was to evaluate the effectiveness of the LMC system using serious games specifically designed for the UL in people with PD in mild-to-moderate stages of the disease. In the experimental group, significant improvements were observed in all post-treatment assessments, except for the BBT on the less affected side. For the control group, statistical significance was observed for the PPT on the more affected side. However, according to the statistical intergroup analysis, significant improvements were found for the PPT on the more affected side and the PPT assembly post-treatment in the experimental group, with an excellent satisfaction and compliance.

Our results suggest an improvement in UL coordination, speed of movements and fine dexterity using the LMC system. These findings are in line with previous studies. Allen et al. [20] showed that PD patients improved UL speed of movements compared to the control group after using the Unity game development software® and measured with the Nine Hole Peg Test (NHPT), considered as a gold standard measure of manual dexterity. The sessions were performed at home, three times a week, for twelve weeks. Two of the games developed in this study (the ‘marshmallow’ game and the ‘chicken’ game) focused on UL movements. These two games were played in the same session and thus the patients played each game twelve times. Participants were provided with auditory and visual feedback during both games to assist them and improve their performance. Upon completion of each game participants received feedback on their overall performance, including information about the number of successes, the number of errors and an overall score. Scores were adjusted according to the level of difficulty, so that higher scores were achieved when playing at a more difficult level. Each game had four levels of difficulty to choose from: easy, medium, hard and extreme.

No differences were observed for the other measures used in this study. This may indicate that 12 sessions of semi-immersive VR using the LMC system and the serious games designed for this study may be insufficient for improving UL grip strength and gross dexterity. However, improvements were found for the experimental group in all post-treatment assessments. These positive results may indicate that LMC could be an interesting tool for the UL rehabilitation of PD patients in the mild to moderate stages of the disease, however further studies are needed with longer training periods and a larger sample size.

To our knowledge, there is a lack of published studies that have used the LMC system or any other markerless motion capture system for training functional UL skills in PD. However, several authors have used these devices in other neurological diseases. Iosa et al. [21] developed a pilot training protocol based on the LMC for stroke rehabilitation. A crossover pilot trial was conducted in which six sessions of 30 min of the LMC system were added to conventional therapy. This trial showed improvements in hand abilities measured using the Abilhand Scale and grasp strength measured using a dynamometer. Our results differ with the aforementioned study by suggesting that the design of the proposed protocol and the intrinsic conditions of the serious games designed do not improve grip muscle strength. Wang et al. [22] measured the improvements in functional abilities using the Wolf Motor Function Test in a sample of stroke patients after a Leap Motion-based VR training compared with conventional therapy. In the experimental group, patients were given Leap Motion-based VR training for 45 min, once a day, five times a week for four weeks, as well as conventional occupational therapy for 45 min, once a day, five times a week for four weeks. In the control group, the patients only received conventional occupational therapy training twice a day, each for 45 min, five times a week for four weeks. Their results showed that both groups obtained significant improvements in the motor function of the affected ULs and in the action performance time, however the improvements were greater in the experimental group. Our results also showed post-treatment improvements on the more affected side. Vanbellingen et al. [23] observed that improvements in dexterity in stroke patients could be due to an intensive, highly repetitive and task-specific training with LMC assessed with NHPT. The intervention consisted of nine 30 min training sessions spread out over a three week period, i.e. three training sessions per week. Our results are line with this study.

The LMC system has also been used as an assessment tool for other motor symptoms of PD, such as tremor. Hironobu and Masashi [24], attempted to measure tremors using the Leap Motion sensor. The purpose was to detect hand motion, which made it possible to measure tremors in the hands without touching them. Chen et al. [25] developed a rapid, objective, and quantitative system for measuring severity of finger tremor to quantify frequency and amplitudes using the LMC system. Butt et al. [26] evaluated motor dysfunction in PD patients, such as slowness of movements, frequency variations, amplitude variations, and speed. In our study, we have not used LMC as an assessment tool for the UL in PD patients. Further studies should include this technology as a quantitative method, in order to provide more accurate parameters for the evaluation of UL motor impairments.

This motion capture rehabilitation method using serious games may be used to treat the UL disorders of PD patients by performing functional exercises in a virtual environment. Moreover, immersive virtual environment attempts to engage the patient to the point of not focusing on the fact of being in a rehabilitation session. Our findings show that the experimental protocol designed for UL rehabilitation in PD is feasible with an excellent satisfaction. Furthermore, all patients completed the protocol with excellent compliance. This is in accordance with other virtual reality studies in which the performance of functional tasks with increasing difficulty and interactive video game environments are shown to enhance motivation and adherence to treatment [9]. These findings, added to the low cost of this semi-immersive VR system, could contribute to the acceptance of this kind of technological treatment as a complementary tool for UL rehabilitation in PD patients.

These results, in terms of the CSQ-8, showed a high level of satisfaction among participants. These data are comparable to Iosa et al. [21] who employed the Pittsburgh Rehabilitation Participation Scale to assess participants’ satisfaction. This study provided a proof of concept that, with a high level of active participation, the LMC system may be a suitable tool, even for elderly patients with subacute stroke. Our results showed an excellent satisfaction with both interventions, with higher values for the LMC treatment.

### Limitations

Although our findings are encouraging, some limitations of our study should be noted. First, the results cannot be generalized for all patients with PD, therefore it is necessary to interpret these findings with caution. Our sample was limited to people with PD in mild-to-moderate stages of the disease. Moreover, the sampling methods could have resulted in a selection bias. Additionally, the use of different outcome measures may have resulted in more significant results (such as NHPT and Action Research Arm Test). Further randomized controlled trials with larger samples, follow up assessment, in order to evaluate side effects, and more intensive dosage are required to verify these results.

## Conclusion

The LMC system and the serious games designed and used in this study represent a rehabilitation tool that may benefit certain PD patients for the improvement of coordination, speed of movements and fine dexterity in UL interventions. This system presents important advantages over other motion capture systems, namely thanks to its portability, ease of use, commercial availability, low cost and non-invasive nature. Future studies are necessary to further research and verify the outcome of this tool and to determine whether there is an ideal patient type who may benefit more from these interventions.

## Data Availability

All the data and materials could be found at Faculty of Health Sciences of Rey Juan Carlos University.

## Abbreviations

BBT: Box and blocks test
CSQ-8: Client Satisfaction Questionnaire
FG: Flip game
GG: Grasp game
LMC: Leap motion controller
NHPT: Nine hole peg test
PD: Parkinson’s disease
PG: Pinch game
PI: Piano game
PPT: Purdue pegboard test
RG: Reach game
SG: Sequence game
UL: Upper limb
VR: Virtual reality
